# A roadmap for strengthening evidence-informed health policy-making in Iran: protocol for a research programme

**DOI:** 10.1186/s12961-019-0455-9

**Published:** 2019-05-17

**Authors:** Haniye Sadat Sajadi, Reza Majdzadeh, Bahareh Yazdizadeh, Farideh Mohtasham, Mahsa Mohseni, Leila Doshmangir, John Lavis

**Affiliations:** 1 National Institute for Health Research, Tehran University of Medical Sciences, Tehran, Iran; 2 Knowledge Utilization Research Centre, Tehran University of Medical Sciences, Tehran, Iran; 3 Tabriz Health Services Management Research Center, Iranian Center of Excellence in Health Management, School of Management and Medical Informatics, Social Determinants of Health Research Center, Health Management and Safety Promotion Research Institute, Tabriz University of Medical Sciences, Tabriz, Iran; 4 McMaster Health Forum and Department of Health Research Methods, Evidence and Impact, McMaster University, Hamilton, Canada

**Keywords:** Evidence-informed health policy-making, evidence-based practice, Iran

## Abstract

**Background:**

Many initiatives have been taken in the Islamic Republic of Iran to promote evidence-informed health policy-making (EIHP). However, these initiatives are not systematic. Since the implementation of EIHP is not consistent and the interventions in this regard are complex, a comprehensive plan could be a useful tool for employing initiatives to achieve and promote EIHP. Hence, this study aims to develop a roadmap for strengthening EIHP over a 3-year period in Iran.

**Methods:**

Nine projects will be conducted to define the roadmap for strengthening EIHP. These projects include two reviews and a stakeholder analysis to identify the factors that facilitate or hinder achieving EIHP. The next study will be a qualitative study to prioritise the challenges and outline the main causes. The following steps will be a review of reviews to extract global experiences on interventions used for strengthening EIHP and two qualitative studies to examine the adoption of these interventions and develop an operational plan for strengthening EIHP in Iran. The research will be completed through conducting two qualitative-quantitative studies to design a tool for measuring EIHP and assessing EIHP in Iran at baseline.

**Discussion:**

This national EIHP roadmap will surely be able to identify the gaps and bumps that might exist in the implementation plan for establishing EIHP and eliminate them as needed in the future. This roadmap can be a step in moving towards transparency and accountability in the health system and as thus towards good governance and improvement of the health system’s performance. Although the plan can be a good model for developing countries and may promote the use of evidence in health policy-making, we should assume that there are some critical contextual factors that could potentially hinder the complete and successful implementation of EIHP. Thus, to enhance EIHP in these countries with a policy-making context that does not fully support the use of evidence, it is crucial to think about not only those interventions that directly address the EIHP barriers, but also some long-term strategies to make required changes in the context, both beyond and within the health system.

## Background

Iran has undertaken a number of important initiatives, particularly during the recent health sector reform (Health Transformation Plan) that began in 2014, to achieve healthcare for everyone by expanding universal health coverage (UHC) by 2025 [1–4]. As a result, the country has witnessed remarkable improvements in access to and coverage of healthcare services for all social classes, especially for the poor [5]. Nevertheless, there have been a number of concerns in Iran’s path towards UHC, such as improving the quality and efficiency of services as well as addressing levels of government coverage of health services, essential medicines and other medical technologies [6]. To tackle these challenges, policy-makers encounter a wide range of policy options. Numerous factors can affect the feasibility and sustainability of these options, including income level, health financing and governance arrangements. To support policy-makers in these decision-making processes, providing scientific evidence plays a major role. Decisions informed by evidence are much stronger and have better potential to convince policy stakeholders on the selected policy option. Evidence-informed health policy-making (EIHP), an approach in which the right answers could be given to policy-makers by providing ‘the right evidence, at the right time and in the right language’ [7], thus preparing them for efficient and effective decision-making, is therefore urgently needed in Iran.

The importance and consequences of EIHP are widely acknowledged in the literature [8–12]. The need to adopt evidence-informed policies, programmes and interventions for the rapid achievement of UHC, particularly in low- and middle-income countries, was clearly emphasised in the 2013 WHO report [13]. The experience of implementing various sector reforms has shown that the probability of achieving universal health coverage and equitable access to healthcare increases when decisions are made based on evidence [14–16]. Nevertheless, the literature tells us that the lack of use or misuse of evidence remains one of the main challenges of health systems across the world [17, 18].

Many initiatives have been conducted to increase and institutionalise the EIHP and to identify its determinants [19–27]. Moreover, a number of interventions have been undertaken at various levels across the world to promote EIHP [20, 22, 24, 25, 28–36]. Some of these interventions have targeted knowledge-producing organisations whereas others have targeted knowledge-utilising organisations. One such intervention is the establishment of knowledge translation platforms, such as the Evidence Informed Policy Networks (EVIPNet) and Getting Research into Policy and Practice (GRIPP) programme, whose goal is to build capacity among researchers, policy-makers and citizens to exchange information and to promote the systematic use of research evidence in health policy-making [37, 38]. However, in spite of these EIHP initiatives, getting evidence into policy in most low- and middle-income countries remains a very challenging task, with huge gaps remaining and the lack of use or misuse of evidence continuing to be one of the main challenges of health systems in these countries [17, 18].

The increasing growth of global attention to the use of evidence in health policies motivated Iran’s health system to introduce some initiatives toward promoting EIHP [39–43]. As a result, especially in recent years, the use of research evidence has been considered in key stages of policy-making processes, including developing, implementing and evaluating policy options. However, due to some barriers of EIHP in Iran’s health system, these efforts are not systematic, comprehensive nor well institutionalised. Since there is a wide range of barriers to EIHP, it seems that there is a need to develop a comprehensive plan for enhancing the use of evidence in policy-making. To develop this plan, the factors that facilitate or hinder achieving EIHP, both currently and in the future, must be identified with the participation of all stakeholders. Thereafter, by prioritising the challenges and outlining the main causes, practical and appropriate interventions should be chosen based on global experiences and the country’s specific circumstances. Their operationalisation should then be organised within a specific financial and temporal framework. Furthermore, to ensure progress of the EIHP approach, it would be better to continuously assess the status quo of EIHP in the country and the impact of interventions. Thus, an appropriate tool must be designed and standardised to evaluate the status of EIHP. Hence, this study protocol is being conducted with the goal of developing a roadmap for strengthening EIHP over a 3-year period (2020–2022). Important questions that need to be answered to achieve this goal are as follows: What are the main barriers and facilitators of EIHP considering the status quo and the future trends affecting the health system? How can the EIHP status be assessed? Which important steps should be taken in order to strengthen EIHP? The results of this study can help institutionalise EIHP in the health sector and be a good model for developing countries.

## Methods/Design

**Fig. 1:**
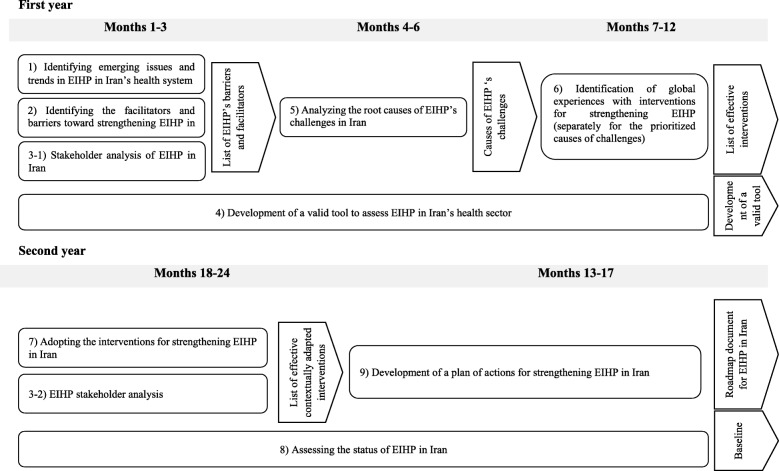
The map for studying efforts to enhance evidence-informed health policy-making in Iran

To develop the roadmap for strengthening EIHP in Iran, nine research projects will be conducted. These projects will be conducted simultaneously in some areas and consecutively in others. The whole project will last approximately 2 years (Fig. 1). What follows is a description of the methods that will be applied in each project.

### Project 1: Identifying emerging issues and the likely barriers and facilitators to support EIHP in the Iran health system

This project is being undertaken to identify the probable barriers and facilitators of EIHP in the health sector through a qualitative approach. The data will be collected in three stages. In the first phase, the research team will define the study questions. These questions must be described as such so that they can cover the topic under study, i.e. identifying the barriers and influential factors that may affect EIHP in the future in Iran. Subsequently, by using the available documents (including the health system situation analysis reports, supplementary reports of the national scientific map and the national health map), the list of issues and trends that may affect the health system in the future will be extracted. These trends and issues will be classified using the STEEP (social, technological, economic, ecologic, and political) model. With the help of this model, information regarding the important future causes of change will be identified in various components, including the social, technological, economic, ecologic and political components, and their outcomes will be classified as opportunities or threats. In the second phase, a focus group discussion (FGD) will be held with relevant experts to outline the main important trends. In the FGD session, in which experts familiar with health policy-making, trends and mega-trends analysis will be attending, the participants will be asked to discuss and exchange ideas on the trends affecting EIHP in Iran. These participants will be informed of the meeting’s objectives beforehand by phone or email. After the initial agreement, invitations will be sent along with a list of trends extracted from the first phase. Two facilitators will be responsible for steering the communications. With the participants’ consent, their conversations will be audio-recorded with a digital voice-recorder. Notes will also be taken during the discussion. After listening to the audio files, they will be transcribed verbatim. A summary of emerging trends and issues identified at the meeting will then be emailed to the participants for their revision and completion if needed. The identified trends will be finalised according to the participants’ comments. In the second FGD session, the participants will be asked to discuss the impact of identified trends and the opportunities and threats faced by EIHP in Iran. At this stage, the same FGD participants will be invited. The summary of the meeting will be compiled in the form of a list of influential trends. The most important future opportunities and trends of EIHP in Iran that were recognised from the identified trends will also be compiled and finalised in a meeting held by the research team. In the third phase, the research team will determine the scenario’s components and share them in group discussions to identify relevant scenarios.

### Project 2: Identifying facilitators and barriers towards strengthening EIHP in Iran

Several studies have been conducted to identify the factors influencing the improvement of EIHP. Therefore, we decided to conduct a systematic review to summarise the results of the available studies. This review will include investigations that have examined the facilitators and barriers to EIHP. The inclusion criteria for the articles will be all research that has identified factors enabling or hindering the use of evidence in health policy at a macro level in Iran. The studies on clinical decision-making and/or service-providing organisations (such as hospitals) will be excluded. The articles will be in both Persian and English. The search will be conducted using appropriate Persian and English keywords in PubMed, Medline, Scopus, Embase, Health Systems Evidence and Scientific Information Database. To increase the sensitivity of the search, hand-searching will be done in relevant national and international journals, reference lists and related documents from various organisations (including the Parliamentary Research Center, Higher Research Institute, the Policy-making Council of the Deputy of Coordination, Deputy of Planning, the Academy of Medicine and governmental legislation), and citation tracking will be performed. Endnote software will be used to manage the references. The primary screening (based on the article title and abstract), the secondary screening (based on the article full texts) and data extraction will be independently conducted by two individuals and, in the case of disagreement, and it will be examined by the research team. The data extracted from each article will be as follows: title, year of publication, year in which the study has been conducted, study objective, methods, population under study, sampling frame and procedure, sample size, data collection tool, analysis method, barriers, facilitators, interventions and outcomes relevant to EIHP, stakeholders mentioned in the study, and the method of evaluating EIHP status. The systematic review will be a mixed research synthesis of the integrated design types [44], and all types of studies (quantitative, qualitative and mixed) will be included.

### Project 3: Stakeholder analysis of EIHP in Iran

A stakeholder is an individual or a group that may affect the organisation and in turn be affected by the organisation’s actions, policies, practices and decisions [45]. Any policy or programme has different stakeholders, some of which may be strong supporters and others may have the power to weaken the programme. Stakeholder analysis is a systematic way to analyse stakeholders’ perspectives on a topic. Since the measures they take may greatly influence the topic, stakeholder analysis is considered a useful tool for planning, assessing a system and potential changes to it [46]. In this study protocol, the stakeholder analysis will be done twice. The first one will be done to determine the key stakeholders’ views regarding the barriers and facilitators to EIHP. To do this, the stakeholders will be identified through a brainstorming session with the research team’s members and a few experts on the topic. The list of identified stakeholders will be categorised based on their role at three activities of knowledge translation, including push, pull and exchange. Then, a FGD will be held to examine the stakeholders’ perspective. In the FGD, the stakeholders will be asked to discuss identified facilitators and barriers to EIHP in Iran. These participants will be informed of the meeting’s objectives beforehand by phone or email. After the initial agreement, invitations will be sent along with a list of identified facilitators and barriers by two previous projects. One facilitator will be responsible for steering the communications. With the participants’ consent, their conversations will be audio-recorded with a digital voice-recorder. Notes will also be taken during the discussion. After listening to the audio files, they will be transcribed verbatim.

The second stakeholder analysis will be conducted to determine whose interests should be taken into account when implementing interventions of the plan. To do this, the key stakeholders of each proposed intervention will be identified through a brainstorming session with the research team’s members. To analyse the stakeholders’ power, position and interest, a list of interviewees will be prepared through purposive and respondent-driven sampling and they will be interviewed. The selection criteria will be based on their knowledge, experience, impact and involvement in EIHP in Iran. We will explore influence and power by questions focusing on the alliances and resources of the stakeholders (e.g. who is in charge, what resources they have and how they can directly or indirectly make an impact). Interviews will be continued until the point of data saturation is reached. An interview consent form will be completed by each interviewee before starting. The qualitative data will be analysed through a thematic analysis (inductive and deductive) approach. Based on interpretation of the interview, stakeholders’ power and influence will be categorised, according to whether they had high or low influence on the intervention, and specifically the degree to which they were able to place the issue on the public or political agenda, influence legislation, or actively participate in implementing and mobilising the intervention. At this stage, the stakeholders’ matrix will be drawn based on their characteristics. Finally, given the position of each stakeholder, appropriate strategies will be designed and presented for better engagement and interaction through a brainstorming session. The output of this stage of the study will be a list of EIHP stakeholders and actors, along with their characteristics and strategies for encouraging their participation.

### Project 4: Development of a tool to assess EIHP in Iran

To assess EIHP status at baseline and post interventions, a tool is needed to properly measure it. There are limited tools for measuring EIHP, most of which subjectively measure policy-maker knowledge, individual and organisational capacity, and the factors affecting EIHP. For example, Lavis et al. [47] and Imani-Nasab et al. [48] introduced tools in 2011 and 2017, respectively, that examine the factors affecting EIHP based on the planned behavioural change theory. Another tool is the checklist ‘Is research working for you? A self-assessment tool and discussion guide for health services management and policy organizations’ provided by the Canadian Foundation for Healthcare Improvement [49], which also measures the individual and organisational infrastructures and capacity in evidence-utilising organisations. What is common among these tools is the evaluation of the policy-makers’ and experts’ perceptions of EIHP. A review of the literature indicates that, although many programmes and models are available for promoting EIHP status, a valid, reliable and local tool for EIHP assessment, and subsequently the monitoring of the changes brought about by corrective measures, is still lacking. Thus, the aim of this project is to design and validate a tool evaluating EIHP in Iran, using objective questions that can be used to determine the status quo and assess the impact of interventions. There are two stages in this project, namely developing the questionnaire and standardising the developed questionnaire. To develop the questionnaire, we will conduct a narrative review (on ‘policy impact’ definitions and a way to measure it) and interview the policy-makers and managers in Iran’s Ministry of Health and Medical Education (MOHME) (to understand their day-to-day work and the type of decisions they make) to decide on suitable constructs and related questions during research team meetings. To standardise the questionnaire, we will perform cognitive interviews to enhance the validity and test retest to assess the reliability of the questionnaire. Eventually, the output of this project will be a valid and reliable contextualised tool for evaluating EIHP in Iran.

### Project 5: Analysing the root causes of EIHP challenges in Iran

Given the outputs of the first and second projects, and upon extracting EIHP’s challenges, this phase will be conducted to identify the major challenges faced by EIHP in Iran using the root cause analysis approach and ‘the 5 Whys technique’. Data collection will take place through FGDs. The participants will be selected purposively from among EIHP stakeholders in Iran, including health policy-makers, directors of various health organisations and faculty members. The participants will be chosen in accordance with the challenges selected for that session. During the session, a list of relevant challenges will be selected and handed out to the participants. First, they will be asked why this issue has arisen. All the responses will be noted down in a flip chart. Gradually, and step by step, other ‘whys’ will be asked and the responses will be written in the flip chart. The method of analysis will be in the form of a tree diagram based on the nature of the identified barriers and challenges. The outcome of this study will be a list of prioritised challenges faced by EIHP in Iran.

### Project 6: Identification of global experiences on the interventions for strengthening EIHP

Selecting the appropriate interventions to strengthen EIHP should be informed by scientific evidence. Thus, the best option is to review global experiences to choose these interventions instead of simply using expert opinions. Therefore, to prepare a list of previously tested interventions to confront highly prioritised EIHP challenges, a review of current reviews [50–53] will be conducted. All reviews that have examined the effectiveness of efforts to confront the challenges of EIHP are eligible to enter. To find eligible reviews, electronic databases and relevant journals will be searched. The search will be done with relevant keywords. Well-known authors will also be contacted. Endnote software will be used to better manage the references. The details of the reviews will be extracted using a form that contains objective, background, research approach, participants, methods of data collection and analysis, main findings and key messages. Meta-synthesis will be used to combine the review results if possible.

### Project 7: Adapting interventions for strengthening EIHP

A good programme should propose context-specific solutions in order to achieve a successful outcome. Thus, this project will aim to provide tailored solutions extracted from previous settings to overcome the identified and prioritised challenges of EIHP. The interventions will be contextualised according to two variables, namely general political and health system characteristics. In the general political environment, political institutions (government structures, policy legacies, stakeholder engagement in policy-making), interests of active political groups, alignment of interventions with beliefs and values, and the effect of external factors (political change, economical change, technological change, new diseases, media coverage) will be considered [54]. In health system characteristics, we will consider how governance, financial and delivery arrangements will affect the adaptation and implementation of interventions. The contextualisation process will be performed by holding FGDs with relevant experts. During these discussions we will attempt to assess the feasibility of the proposed solutions by stating the challenges faced by EIHP in Iran along with the global knowledge for confronting these challenges. The output of FGDs will be a policy brief on contextualised adapted solutions for strengthening EIHP in the country. The policy brief will be presented in a policy dialogue with relevant experts and actors.

### Project 8: Assessing the status of EIHP in Iran

Based on reports and expert opinion, the status of EIHP in Iran remains far from desirable [55]; further, there is inadequate data on its various aspects, including its strengths and weaknesses. Given the significance of assessment in determining the effectiveness of interventions, outlining the strengths and weaknesses and helping to design and propose solutions for improvement, an assessment of EIHP is necessary to determine the baseline for systematic comparisons. Using the standardised tool in project 4, EIHP will be assessed at the macro level of policy-making, i.e. MOHME. To this end, a quantitative study will be executed using the tool developed in the previous study project.

The context of the study is Iran’s health sector. Since the tool applied has not yet been finalised, we cannot determine the details of the size and characteristics of the sample. However, it seems that the respondents will be selected from the MOHME’s senior managers (including all the Ministry’s Deputies and all their subdivisions), health policy-makers in law-making bodies (Islamic Parliament, Expediency Council, and Supreme Council of the Cultural Revolution) and health policy-makers in resource allocation organisations (Planning and Budget Organisation, National Treasury). The method of analysis will be selected based on the type of tool employed.

### Project 9: Development of a plan of action for strengthening EIHP in Iran

The development of a plan of action can effectively steer interventions towards targeted relevant efforts for strengthening the status quo and abstaining from wasting of resources. We expect that the development and utilisation of this plan will help bring about effective results for the health system and improve its governance. To conduct this project, first the programme development framework will be finalised by examining the existing frameworks and relevant elements by the research team, including the activities, timeframe, person responsible and the budget. Given the outputs of earlier projects, each of which covers an important section of the main elements of the programme, an operational plan for strengthening EIHP will be developed for Iran’s health sector. Finally, a skill-based capacity-building programme will be tailored to the needs of each stakeholder.

## Discussion

While Iran’s government has committed to achieve UHC [56], its health system faces several challenges[57]. These challenges have to be appropriately addressed through tough decisions made by health policy-makers[58]. While it can be seen as a threat for our health system, we think it is a good opportunity to strengthen EIHP as a key element of good governance and a means to improve health systems performance. To our knowledge, most initiatives performed to improve EIHP in Iran focus on issues of access to evidence or building individual capacity [59]. However, previous studies conducted in Iran have shown that the main barriers of EIHP are multiple and related to the complex decision-making environment [55]. Thus, it is a must to choose a number of system-level interventions that address these barriers, aiming to change decision-making processes to support the appropriate use of evidence. This is why we are interested in developing a comprehensive plan for strengthening EIHP in the country.

Although the proposed roadmap may improve the use of evidence in health policy-making, we presume that there are some critical factors that hinder the full and successful implementation of EIHP. Contextual factors are an example of the barriers that can affect the use of evidence in health policy-making. With a shortage of overarching transparency and accountability, it is hard to expect that we can make sustainable changes to improve the use of evidence in decision-making and to institutionalise this approach. Because of this, the changes in these factors are out of the scope of the present project and cannot be achieved in the short term. On the other hand, they largely determine the ways in which the evidence is used in the policy process, as well as being a criterion for what should be considered to be good evidence. Of course, political instability can affect the situation. Therefore, conflicts with other countries and geo political challenges can change EIHP in a significant way.

We also assume that our plan will be at risk of a conflict of interest and the process employed to achieve an impact in policy-making. There are many different stakeholders with varied and incompatible interests and aims. The conflict that they have in their interests can impact both what constitutes evidence and how the evidence is interpreted and applied. They can also bring personal issues or relationships to the table that might not be addressed, altering the purpose and context for decision-making. In conclusion, to enhance EIHP in countries with policy-making contexts that do not fully support the use of evidence, it is crucial to think about not only those interventions that directly address EIHP barriers, but also long-term strategies necessary to implement the required changes both beyond and within the health system.

## Abbreviations

EIHP: evidence-informed health policy-making
FGD: focus group discussion
HSE: health systems evidence
MOHME: Ministry of Health and Medical Education
UHC: universal health coverage
